# Supplementary material for: Collation of a century of soil invertebrate abundance data suggests long-term declines in earthworms but not tipulids
Source: PLoS One. 2023 Apr 3;18(4):e0282069. doi: 10.1371/journal.pone.0282069 (PMC10069791; doi:10.1371/journal.pone.0282069)
Supplement: S1 Table — (DOCX) [file pone.0282069.s001.docx]

| **Section and Topic** | **Item #** | **Checklist item** | **Location where item is reported** |
| --- | --- | --- | --- |
| **TITLE** | | |  |
| Title | 1 | Collation of a century of soil invertebrate abundance data suggests long-term declines in earthworms but not tipulids. |  |
| **ABSTRACT** | | |  |
| Abstract | 2 | See Abstract of paper |  |
| **INTRODUCTION** | | |  |
| Rationale | 3 | Large-scale declines have been recorded in a number of invertebrate groups. There has been no assessment of potential long-term abundance changes in soil invertebrates due to a lack of long-term monitoring data. Many invertivorous bird species have declined particularly in farmland and woodland, which suggests that the invertebrates they rely on may also be declining. To address this, we collate historical soil invertebrate abundance data from previously published studies to assess whether these data may be used to infer changes through time, and to assess whether those changes vary between habitats. |  |
| Objectives | 4 | Our aims are to assess, firstly, if it is possible to identify differences in soil invertebrate abundance between habitats and through time and, secondly, to test the following specific hypotheses: 1) that soil invertebrates, particularly earthworms and tipulids, have declined in the UK and 2) that those trends vary between habitats. |  |
| **METHODS** | | |  |
| Eligibility criteria | 5 | This paper seeks to identify papers that contained relevant soil invertebrate abundance data from field studies in the UK in a repeatable manner with some meta-analysis on the collated data, but does not attempt to be a full systematic review. | Methods |
| Information sources | 6 | BTO Library and Google Scholar Dec 2019/Jan 2020  IBIS, Functional Ecology and Journal of Ecology May 2020  Animal Ecology and Insect Conservation July 2020  Applied Ecology, Bird Study and Biological Conservation September 2020  Ethos May 2021  Citations throughout 2020 and 2021 | Flow Diagram |
| Search strategy | 7 | Manual searching of contents pages of the following journals identified as those most likely to contain relevant data from the UK: IBIS, Journal of Animal Ecology, and started Journal of Applied Ecology, Journal of Ecology, Functional Ecology, Insect Conservation. We then compared the efficacy of manual searching of contents pages with keyword searching (see paper results) and applied keyword searches to the following additional journals most likely to contain relevant data from the UK: Bird Study, Biological Conservation, Journal of Zoology and finished searching the rest of Journal of Ecology, Functional Ecology, Insect Conservation. We also used the same keywords to search the Ethos British Library database of UK university theses given their potential to also contain useable data. Apparently relevant papers that were referenced by eligible studies identified by these searches were also considered for inclusion in the review using the same criteria. | Methods |
| Selection process | 8 | Papers were selected independently by six reviewers, with the screening carried out by one reviewer (AB) discussed with a second reviewer (JPH) as required to make sure that each entry was selected correctly and data extracted. Papers were included if they contained relevant data on soil invertebrates that could be extracted. No automation tools were used in this process. | Methods |
| Data collection process | 9 | An independent reviewer collected/extracted the data (AB) which was checked by a second reviewer (JPH). No automation tools were used in this process. All data was collected into a bespoke Excel spreadsheet. |  |
| Data items | 10a | Data outcomes: each study should contain UK data on the abundance or biomass of a clearly defined group of soil invertebrates. Variables extracted and included in the database are: reference, year start and end, habitat, location, country, geography, method of extraction, sample size, area, diameter and depth of sample, month/season sample was taken, finer location and habitat details, the units the study presented the data in for accurate conversion, and numbers of soil invertebrates. |  |
|  | 10b | Listed above, but where data is missing additional sources were researched and if none found studies were not included in the analysis (approx. 10 studies). Another approx. 17 studies were lacking specific variable data, e.g. sample sizes, depth, which were sought and retrieved from previous publications. Where the total numbers of soil invertebrates was missing the calibration was used to estimate these from biomass or the total number of adults. If sampling date was missing, then we assumed this was 2 years pre publication. Some papers were excluded from the finer-scale analysis of habitat or method if insufficient information was provided, the impact of which is described in the paper. |  |
| Study risk of bias assessment | 11 | A mixed effects model was applied that took into account study identity and location as random factors. Each model was run with and without a weighting of sample extent (area of the sample multiplied by the number of samples) to account for potential bias in sampling effort for each study. Other analyses were also presented in the appendices to account for potential changes in sample extent (Appendix 1) and study type (Appendix 2) that may have occurred through time. One reviewer assessed each study independently but together with a second reviewer for any complicated studies. |  |
| Effect measures | 12 | Extracted measures were of earthworm or tipulid density and biomass (see methods). |  |
| Synthesis methods | 13a | As above, studies were used that contained relevant soil invertebrate data, with the majority of the data collated from earthworms and tipulids. |  |
|  | 13b | As described in the methods of the paper, missing abundance data could be estimated if biomass or adult earthworm data were presented, using calibration equations. We also standardised the reporting of abundances to the number of individuals per square metre for analysis. | Methods |
|  | 13c | All results were entered into an Excel spreadsheet. | Methods |
|  | 13d | Analysis was used to assess whether numbers of earthworms and tipulids had changed over time and if this varied with habitat – R package glmmTMB was used to apply generalised linear mixed effects model. |  |
|  | 13e | We used mixed effects to account for the non-independence of data between studies and locations. We tested for variation in abundances with year and habitat, and tested for interactions between the two to test for heterogeneity in trends between habitats. |  |
|  | 13f | Studies varied widely in sample extent, so we presented results which accounted for this using a weighting variable, or that did not. We used the concordance between the two models as a test of model robustness. Similarly, studies varied in the methods used to estimate earthworm and tipulid abundance which we accounted for in the models using two different resolutions of detail. |  |
| Reporting bias assessment | 14 | Study location and study identity were used as random effects to account for the non-independence of data from the same study / location. We used sample extent as a weighting variable in one analysis to give more weight to studies with more extensive data. | Appendix |
| Certainty assessment | 15 | The studies used were all published in reputable journals with peer review or Ethos after having gone through assessment for thesis production. A number of different models were produced to test for potential impacts of variation in field method, sample extent and study type. | Methods – weighting |
| **RESULTS** | | |  |
| Study selection | 16a | The number of papers scanned using a manual search of the contents pages of the following journals:  IBIS (1936-2018) – 4821 papers  Animal Ecology (1948-2009) – 4097  Applied Ecology (1960-2002) – 3011  J. of Ecology (1990-2006) – 1492  Functional Ecology (1997-2006) – 1127  Insect Conservation (1997-2003) – 186.  The number of papers identified from keyword search, screened, and included are shown in the flow diagram Figure S1. | Flow |
|  | 16b | A number of studies were excluded either as they sampled invertebrates as prey for other taxa, e.g. in faecal samples or stomach contents, were laboratory experiments, in marine environments or grouped all soil invertebrates together in the results, as in the case of: Green, R. (1988) Effects of Environmental Factors on the Timing and Success of Breeding of Common Snipe Gallinago gallinago (Aves: Scolopacidae). *Journal of Applied Ecology,* *25*(1), 79-93. |  |
| Study characteristics | 17 | Please see supplementary material as too many studies to include here neatly. |  |
| Risk of bias in studies | 18 | See above supplementary material. |  |
| Results of individual studies | 19 | See supplementary material mentioned above. |  |
| Results of syntheses | 20a | See supplementary material mentioned above | Discussion |
|  | 20b | See results, supplementary material and Appendices 1 and 2. | Results |
|  | 20c | See results, supplementary material and Appendices 1 and 2. |  |
|  | 20d | See results, supplementary material and Appendices 1 and 2. |  |
| Reporting biases | 21 | N/A | N/A |
| Certainty of evidence | 22 | See comparison of different models in the results, supplementary material and Appendix 2. |  |
| **DISCUSSION** | | |  |
| Discussion | 23a | See discussion. | Discussion |
|  | 23b | See discussion. | Discussion |
|  | 23c | See discussion. | Discussion |
|  | 23d | See discussion. | Discussion |
| **OTHER INFORMATION** | | |  |
| Registration and protocol | 24a | This review was not registered as it is not an official Systematic Review or Mapping exercise. |  |
|  | 24b | A protocol was not prepared. |  |
|  | 24c | N/A | N/A |
| Support | 25 | Funding from Major Donors Simon Cook and Gillian & Justin Wills, the Penchant Foundation, and a Gift in Will from Kenneth Trouth. |  |
| Competing interests | 26 | No competing interests from the authors involved. |  |
| Availability of data, code and other materials | 27 | Template data collection forms: See supplementary material database.  Data extracted from included studies: See supplementary material database.  Data used for all analyses: See supplementary material database. | Supplementary Material |

*From:*  Page MJ, McKenzie JE, Bossuyt PM, Boutron I, Hoffmann TC, Mulrow CD, et al. The PRISMA 2020 statement: an updated guideline for reporting systematic reviews. BMJ 2021;372:n71. doi: 10.1136/bmj.n71

For more information, visit: <http://www.prisma-statement.org/>
