# Supplementary material for: Collation of a century of soil invertebrate abundance data suggests long-term declines in earthworms but not tipulids
Source: PLoS One. 2023 Apr 3;18(4):e0282069. doi: 10.1371/journal.pone.0282069 (PMC10069791; doi:10.1371/journal.pone.0282069)
Supplement: S1 Fig — (DOCX) [file pone.0282069.s002.docx]

**Identification of studies via other methods**

**Identification of studies via databases and registers**

Records identified from:

Websites: Ethos (n = 3198)

BTO Library (n = 550)

Manual journal searches/scanning:

IBIS (n = 4821),

Animal Ecology (n = 4097),

Applied Ecology (n = 3011),

J. of Ecology (n= 1492),

Functional Ecology (n = 1127), Insect Cons (n = 186)

Records identified through keyword searches from specific journals (n = 4310):

Bird Study (n = 833),

Applied Ecology (n = 3198),

Bio. Cons. (n = 786),

Zool. (n = 3)

Records removed *before screening*:

Duplicate records removed

(n = 90)

**Identification**

Reasons for exclusion include:

no access, laboratory experiment only, lack of field data on soil invertebrates, not from the UK, but # records assigned to each reason not recorded.

Records screened / retrieved

(n = 4220)

Reports sought for retrieval (n = 318):

BTO Library (n = 6)

Websites: Ethos (n = 151)

Manual searches: IBIS (n = 23), Animal Ecology (n = 79),

J. of Ecology (n= 0),

Functional Ecology (n = 1),

Insect Cons (n = 8)

Additional references identified from citations in other records (n = 44)

**Screening**

Studies/Reports assessed for eligibility

(n = 539)

Reports excluded:

Other Species (n = 324)

No Relevant Data (n = 111)

**Included**

Studies included in review (n = 104)

*From:*  Page MJ, McKenzie JE, Bossuyt PM, Boutron I, Hoffmann TC, Mulrow CD, et al. The PRISMA 2020 statement: an updated guideline for reporting systematic reviews. BMJ 2021;372:n71. doi: 10.1136/bmj.n71. For more information, visit: <http://www.prisma-statement.org/>
