# Supplementary material for: Collation of a century of soil invertebrate abundance data suggests long-term declines in earthworms but not tipulids
Source: PLoS One. 2023 Apr 3;18(4):e0282069. doi: 10.1371/journal.pone.0282069 (PMC10069791; doi:10.1371/journal.pone.0282069)
Supplement: S1 File — (DOCX) [file pone.0282069.s003.docx]

## Supplementary Material

### Supplementary Tables

***Table S2. Literature search results.*** *The numbers of papers collated from each journal and the number of papers with data used in earthworm (EW) and tipulid (Tip) analyses.*

| **Journal** | **No. of Papers Possibly Relevant** | **No. of Papers Extracted** | **No. of Papers Used in EW Analysis** | **No. of Papers Used in Tip Analysis** |
| --- | --- | --- | --- | --- |
| Theses | 151 | 34 | 30 | 7 |
| IBIS | 23 | 6 | 5 | 6 |
| Bird Study | 48 | 2 | 3 | 1 |
| Animal Ecology | 79 | 15 | 12 | 4 |
| Applied Ecology | 170 | 25 | 18 | 3 |
| Functional Ecology | 1 | 0 | 0 | 0 |
| Insect Conservation | 8 | 0 | 0 | 0 |
| Zoology | 2 | 1 | 1 | 0 |
| Biological Conservation | 13 | 1 | 1 | 0 |
| OTHER | 44 | 27 | 20 | 8 |
| **TOTAL** | **539** | **111** | **90** | **29** |

***Table S3. Model 5 estimates.*** *Estimates for unweighted and weighted (by natural log of Sample Extent + 1) Model 5 with Fine_Method and Broad_Habitat, compared to the base of Farmland, Autumn and Core. The level of significance is represented by . p < 0.1; * p < 0.05; ** p < 0.01; *** p < 0.001, SE = Standard Error.*

| **Variable Category** | **Variable** | **Unweighted** | | | **Weighted** | | |
| --- | --- | --- | --- | --- | --- | --- | --- |
|  |  | **Estimate** | **SE** | **P** | **Estimate** | **SE** | **P** |
|  | (Intercept) | **5.772** | **0.217** | **<0.001 (***)** | **5.965** | **0.224** | **<0.001 (***)** |
|  | year | **-0.021** | **0.006** | **0.001 (**)** | **-0.019** | **0.007** | **0.004 (**)** |
| *Broad_Habitat* | Human | 0.071 | 0.265 | 0.788 | -0.050 | 0.244 | 0.837 |
|  | Unenclosed | 0.002 | 0.123 | 0.986 | **-0.850** | **0.162** | **<0.001 (***)** |
|  | Woodland | -0.219 | 0.147 | 0.138 | **-0.371** | **0.134** | **0.006 (**)** |
| *Season* | Multiple | 0.077 | 0.149 | 0.605 | **0.297** | **0.118** | **0.012 (*)** |
|  | Spring | 0.054 | 0.063 | 0.395 | 0.076 | 0.060 | 0.210 |
|  | Summer | **-0.352** | **0.071** | **<0.001 (***)** | -0.104 | 0.070 | 0.137 |
|  | Winter | -0.011 | 0.074 | 0.884 | 0.042 | 0.080 | 0.602 |
| *Fine_Method* | Both_Core and mustard | -0.282 | 0.383 | 0.461 | -0.240 | 0.390 | 0.538 |
|  | Surface_Formalin | **-1.004** | **0.180** | **<0.001 (***)** | **-1.300** | **0.187** | **<0.001 (***)** |
|  | Surface_Mustard | **-1.452** | **0.387** | **<0.001 (***)** | **-1.731** | **0.397** | **<0.001 (***)** |
|  | Surface_Permanganate | **-1.955** | **0.208** | **<0.001 (***)** | **-1.678** | **0.200** | **<0.001 (***)** |
|  | Depth | 0.009 | 0.006 | 0.124 | -0.008 | 0.005 | 0.114 |

***Table S4. Model 7 output*** *for unweighted and weighted (by natural log of Sample Extent + 1) Fine_Method with the interaction of Broad_Habitat and year, compared to the base of Farmland, Autumn and Core. The level of significance is represented by . p < 0.1; * p < 0.05; ** p < 0.01; *** p < 0.001, SE = Standard Error.*

|  | **Variable** | **Unweighted** | | | **Weighted** | | |
| --- | --- | --- | --- | --- | --- | --- | --- |
|  |  | **Estimate** | **SE** | **P** | **Estimate** | **SE** | **P** |
|  | (Intercept) | **5.818** | **0.225** | **<0.001 (***)** | **5.936** | **0.225** | **<0.001 (***)** |
|  | year | **-0.022** | **0.007** | **0.001 (***)** | **-0.019** | **0.007** | **0.006 (**)** |
| *Broad_Habitat* | Human | 0.290 | 0.705 | 0.680 | -0.037 | 0.731 | 0.959 |
|  | Unenclosed | **-0.533** | **0.266** | **0.045 (*)** | **-0.944** | **0.177** | **<0.001 (***)** |
|  | Woodland | 0.392 | 0.351 | 0.265 | 0.183 | 0.272 | 0.500 |
| *Season* | Multiple | 0.096 | 0.149 | 0.521 | **0.302** | **0.118** | **0.010 (**)** |
|  | Spring | 0.061 | 0.063 | 0.332 | 0.081 | 0.060 | 0.181 |
|  | Summer | **-0.348** | **0.071** | **<0.001 (***)** | -0.102 | 0.070 | 0.144 |
|  | Winter | -0.008 | 0.074 | 0.917 | 0.043 | 0.080 | 0.594 |
| *Fine_Method* | Both_Core and mustard | -0.237 | 0.379 | 0.532 | -0.208 | 0.384 | 0.587 |
|  | Surface_Formalin | **-1.059** | **0.180** | **<0.001 (***)** | **-1.321** | **0.186** | **<0.001 (***)** |
|  | Surface_Mustard | **-1.353** | **0.384** | **<0.001 (***)** | **-1.652** | **0.392** | **<0.001 (***)** |
|  | Surface_Permanganate | **-1.993** | **0.208** | **<0.001 (***)** | **-1.676** | **0.201** | **<0.001 (***)** |
|  | Depth | 0.008 | 0.006 | 0.138 | -0.008 | 0.005 | 0.113 |
| *Broad_Habitat* Specific Trends | Human | -0.007 | 0.017 | 0.669 | -0.003 | 0.017 | 0.876 |
|  | Unenclosed | **0.022** | **0.010** | **0.024 (*)** | 0.010 | 0.008 | 0.190 |
|  | Woodland | -0.020 | 0.010 | 0.054 (.) | **-0.019** | **0.008** | **0.018 (*)** |

***Table S5. Model 6 estimates.*** *Estimates from the unweighted and weighted (by natural log of Sample Extent + 1) Model 6 containing both Fine_Method and Fine_Habitat variables, compared to the base of Farmland_Arable, Autumn and Core. The level of significance is represented by . p < 0.1; * p < 0.05; ** p < 0.01; *** p < 0.001, SE = Standard Error.*

|  | **Variable** | **Unweighted** | | | **Weighted** | | |
| --- | --- | --- | --- | --- | --- | --- | --- |
|  |  | **Estimate** | **SE** | **P** | **Estimate** | **SE** | **P** |
|  | (Intercept) | **5.543** | **0.232** | **<0.001 (***)** | **5.628** | **0.246** | **<0.001 (***)** |
|  | year | **-0.019** | **0.006** | **0.002 (**)** | **-0.017** | **0.007** | **0.013 (*)** |
| *Fine_ Habitat* | Woodland_Broadleaved | 0.222 | 0.191 | 0.245 | 0.319 | 0.189 | 0.092 |
|  | Farmland_Grass | -0.009 | 0.128 | 0.944 | -0.007 | 0.140 | 0.958 |
|  | Unenclosed_Grassland | 0.194 | 0.168 | 0.250 | -0.189 | 0.206 | 0.358 |
|  | Human_Greenspace | 0.548 | 0.280 | 0.051 (.) | 0.260 | 0.258 | 0.313 |
|  | Human_Industrial | **-1.316** | **0.329** | **<0.001 (***)** | **-1.399** | **0.378** | **<0.001 (***)** |
|  | Farmland_Mixed | 0.134 | 0.485 | 0.782 | 0.184 | 0.333 | 0.580 |
|  | Woodland_Mixed/Conifer | **-0.425** | **0.210** | **0.043 (*)** | **-0.672** | **0.185** | **<0.001 (***)** |
|  | Unenclosed_Moorland | **0.539** | **0.190** | **0.004 (**)** | **-1.435** | **0.291** | **<0.001 (***)** |
|  | Farmland_Pasture | **0.344** | **0.117** | **0.003 (**)** | **0.479** | **0.129** | **<0.001 (***)** |
|  | Woodland_Scrub | 0.153 | 0.574 | 0.790 | 0.121 | 1.027 | 0.906 |
|  | Unenclosed_Wetland | -0.139 | 0.277 | 0.616 | -0.662 | 0.586 | 0.259 |
| *Season* | Multiple | 0.084 | 0.147 | 0.566 | **0.302** | **0.116** | **0.009 (**)** |
|  | Spring | 0.056 | 0.062 | 0.367 | 0.070 | 0.059 | 0.240 |
|  | Summer | **-0.340** | **0.070** | **<0.001 (***)** | -0.105 | 0.069 | 0.128 |
|  | Winter | 0.002 | 0.073 | 0.977 | 0.048 | 0.079 | 0.548 |
| *Fine_ Method* | Both_Core and mustard | -0.339 | 0.377 | 0.368 | -0.222 | 0.387 | 0.566 |
|  | Surface_Formalin | **-0.951** | **0.177** | **<0.001 (***)** | **-1.248** | **0.185** | **<0.001 (***)** |
|  | Surface_Mustard | **-1.556** | **0.380** | **<0.001 (***)** | **-1.762** | **0.393** | **<0.001 (***)** |
|  | Surface_Permanganate | **-1.974** | **0.206** | **<0.001 (***)** | **-1.636** | **0.197** | **<0.001 (***)** |
|  | Depth | 0.010 | 0.005 | 0.068 (.) | -0.006 | 0.005 | 0.194 |

***Table S6. Model 8 estimates.*** *Estimates from the unweighted and weighted (by natural log of Sample Extent +1) Model 8 containing both Fine_Method and Fine_Habitat variables, compared to the base of Farmland_Arable, Autumn and Core. The level of significance is represented by . p < 0.1; * p < 0.05; ** p < 0.01; *** p < 0.001, SE = Standard Error.*

| **Variable Category** | **Variable** | **Unweighted** | | | **Weighted** | | |
| --- | --- | --- | --- | --- | --- | --- | --- |
|  |  | **Estimate** | **SE** | **P** | **Estimate** | **SE** | **P** |
|  | (Intercept) | **5.273** | **0.279** | **<0.001 (***)** | **5.428** | **0.270** | **<0.001 (***)** |
|  | year | -0.011 | 0.008 | 0.148 | -0.008 | 0.008 | 0.274 |
| *Fine_ Habitat* | Woodland_Broadleaved | **1.450** | **0.422** | **<0.001 (***)** | **1.309** | **0.366** | **<0.001 (***)** |
|  | Farmland_Grass | 0.301 | 0.220 | 0.171 | 0.239 | 0.170 | 0.161 |
|  | Unenclosed_Grassland | 0.071 | 0.362 | 0.844 | -0.126 | 0.272 | 0.643 |
|  | Human_Greenspace | **2.322** | **0.790** | **0.003 (**)** | **1.951** | **0.787** | **0.013 (*)** |
|  | Human_Industrial | -1.532 | 1.301 | 0.239 | **-3.336** | **1.448** | **0.021 (*)** |
|  | Farmland_Mixed | -1.291 | 1.327 | 0.331 | **-2.877** | **0.926** | **0.002 (**)** |
|  | Woodland_Mixed/Conifer | **-1.346** | **0.610** | **0.027 (*)** | **-1.256** | **0.449** | **0.005 (**)** |
|  | Unenclosed_Moorland | 0.422 | 0.434 | 0.331 | **-1.286** | **0.336** | **<0.001 (***)** |
|  | Farmland_Pasture | **0.682** | **0.236** | **0.004 (**)** | **0.632** | **0.205** | **0.002 (**)** |
|  | Woodland_Scrub | 0.932 | 2.295 | 0.685 | 2.225 | 3.082 | 0.470 |
|  | Unenclosed_Wetland | -0.218 | 0.466 | 0.640 | -0.539 | 0.584 | 0.356 |
| *Season* | Multiple | 0.121 | 0.147 | 0.410 | **0.321** | **0.116** | **0.006 (**)** |
|  | Spring | 0.054 | 0.062 | 0.389 | 0.061 | 0.059 | 0.302 |
|  | Summer | **-0.349** | **0.070** | **<0.001 (***)** | -0.116 | 0.069 | 0.091 (.) |
|  | Winter | -0.002 | 0.072 | 0.977 | 0.036 | 0.079 | 0.650 |
| *Fine_ Method* | Both_Core and mustard | -0.404 | 0.373 | 0.278 | -0.303 | 0.382 | 0.428 |
|  | Surface_Formalin | **-1.017** | **0.177** | **<0.001 (***)** | **-1.246** | **0.183** | **<0.001 (***)** |
|  | Surface_Mustard | **-1.533** | **0.378** | **<0.001 (***)** | **-1.751** | **0.391** | **<0.001 (***)** |
|  | Surface_Permanganate | **-2.081** | **0.205** | **<0.001 (***)** | **-1.688** | **0.196** | **<0.001 (***)** |
|  | Depth | 0.009 | 0.005 | 0.094 (.) | -0.007 | 0.005 | 0.184 |
| *Fine_ Habitat* Interactions | Woodland_Broadleaved | **-0.042** | **0.012** | **<0.001 (***)** | **-0.044** | **0.012** | **<0.001 (***)** |
|  | Farmland_Grass | -0.009 | 0.005 | 0.097 (.) | **-0.011** | **0.004** | **0.016 (*)** |
|  | Unenclosed_Grassland | 0.009 | 0.012 | 0.461 | 0.004 | 0.010 | 0.724 |
|  | Human_Greenspace | **-0.045** | **0.019** | **0.016 (*)** | **-0.044** | **0.018** | **0.014 (*)** |
|  | Human_Industrial | 0.009 | 0.037 | 0.798 | 0.055 | 0.040 | 0.172 |
|  | Farmland_Mixed | 0.039 | 0.034 | 0.252 | **0.068** | **0.021** | **0.001 (**)** |
|  | Woodland_Mixed_Conifer | 0.024 | 0.016 | 0.141 | 0.012 | 0.011 | 0.295 |
|  | Unenclosed_Moorland | 0.008 | 0.015 | 0.599 | -0.025 | 0.013 | 0.061 (.) |
|  | Farmland_Pasture | -0.009 | 0.006 | 0.112 | -0.006 | 0.006 | 0.274 |
|  | Woodland_Scrub | -0.029 | 0.080 | 0.715 | -0.094 | 0.120 | 0.435 |
|  | Unenclosed_Wetland | 0.015 | 0.017 | 0.378 | 0.015 | 0.022 | 0.488 |

### Supplementary Figures


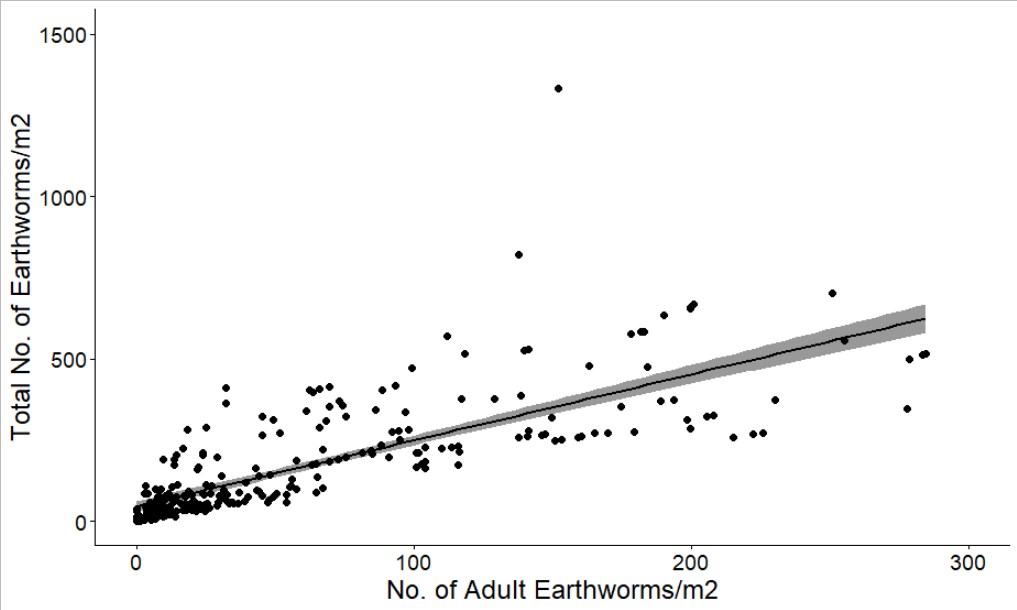


***Fig S2. Calibration curve adult earthworms*** *per m^2^ vs total earthworm abundance per m^2^.*


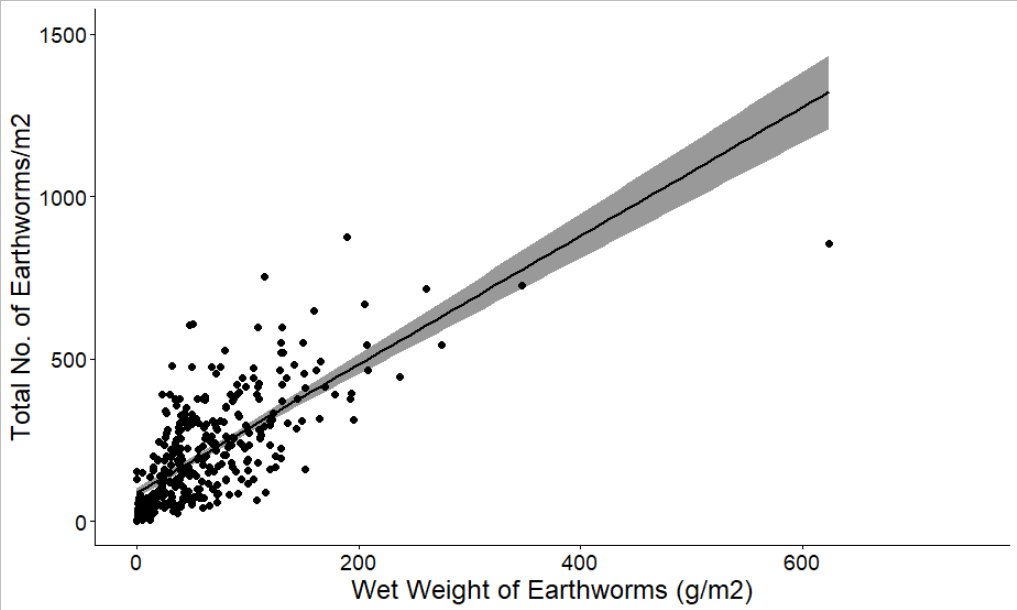


***Fig S3. Calibration curve earthworm wet weight*** *grams per m^2^ vs total earthworm abundance per m^2^.*


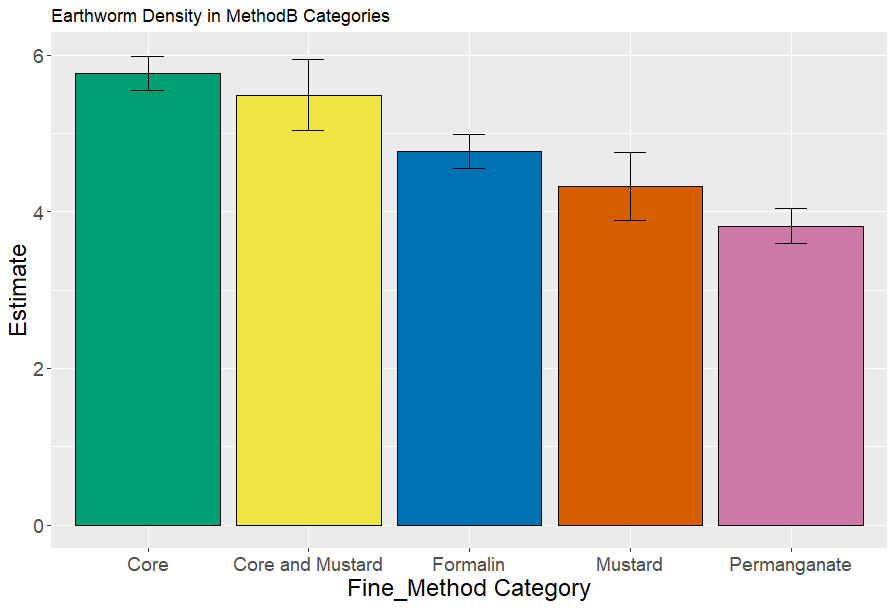


***Fig S4.*** ***Model 5 Fine_Method estimates.*** *Average estimates of logged earthworm density per m^2^ for each Fine_Method category over the study period (Model 5 unweighted). See Table S3 for estimates.*


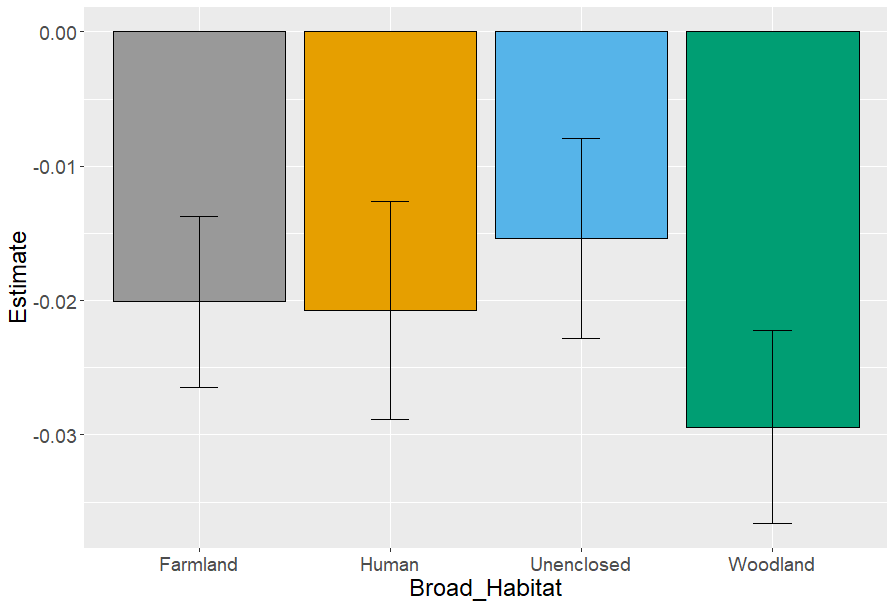


** * * ***

***Fig S5.*** ***Model 7 Broad_Habitat trend estimates.*** *Earthworm density trend estimates in the four Broad_Habitat categories using Fine_Method Model 7 (unweighted). See Table S4 for estimate values (the stars represent significance: * <0.01, ** <0.001, *** <0.0001).*


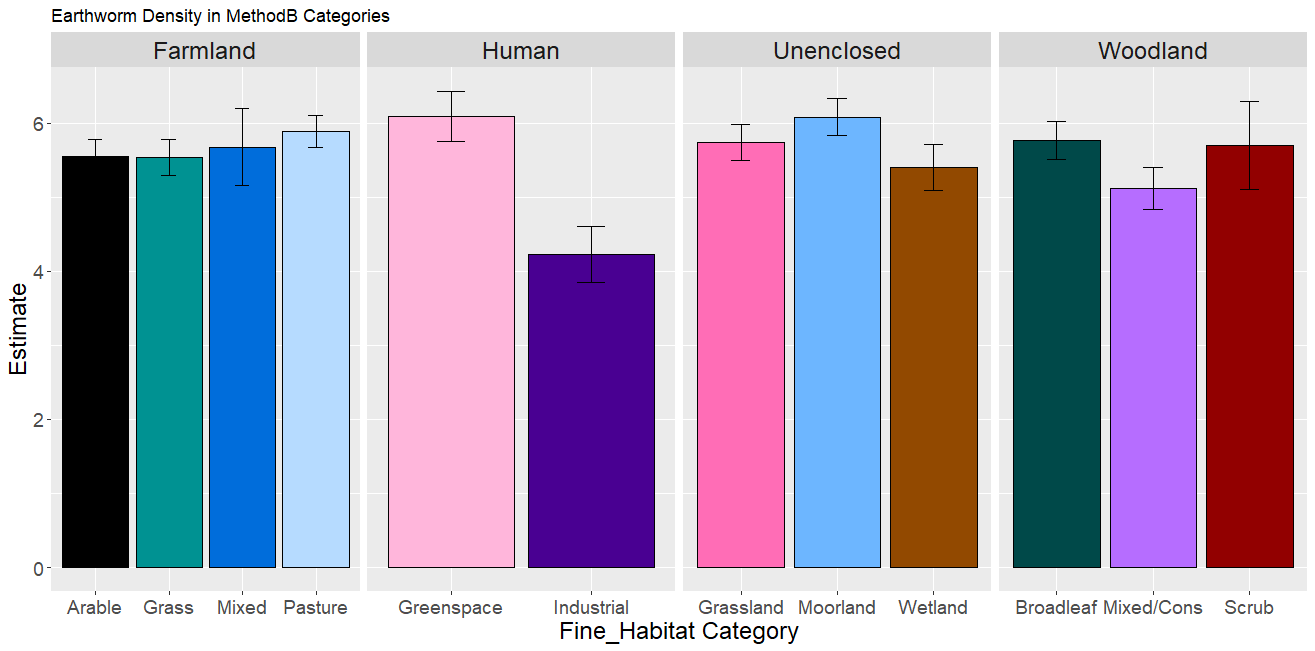
***Fig S6. Model 6 Fine_Habitat estimates.*** *The average estimates over the study period for each Fine_Habitat category, split into the Broad_Habitat categories, using Fine_Method in unweighted Model 6 (see Table S5 for estimate values).*


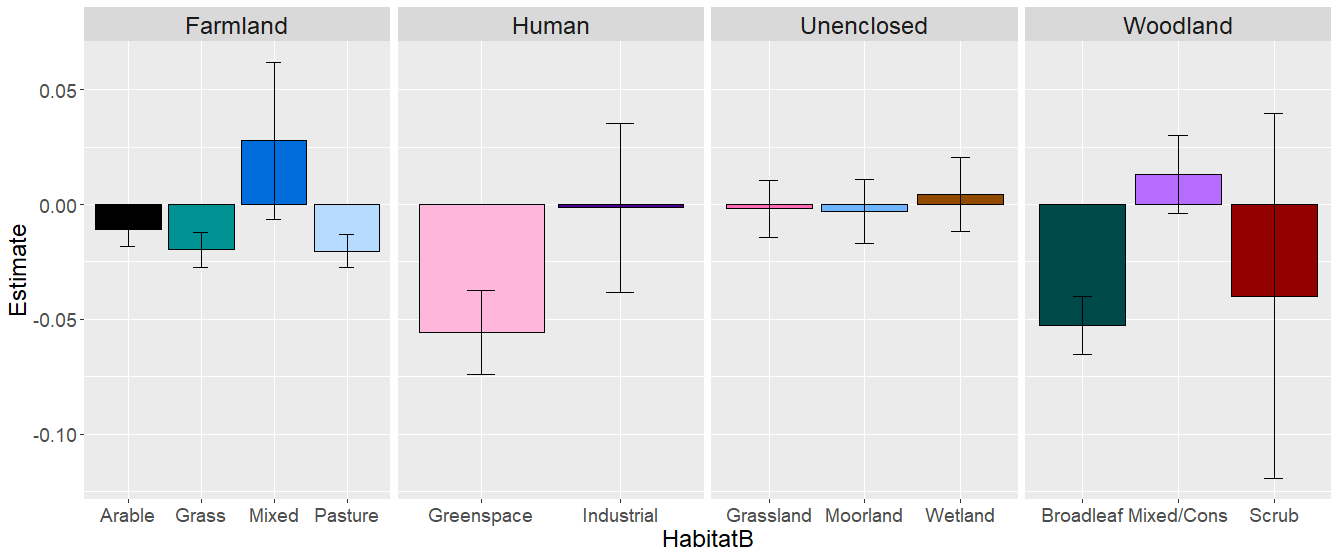


. * ***

***Fig S7. Model 8 trends*** *for each Fine_Habitat category using Fine_Method in the model. See Table S6 for estimate values (the stars represent significance: . <0.1, * <0.01, ** <0.001, *** <0.0001).*

## Appendices

### Appendix 1: Changes in sample extent through time

To check whether there were changes in the extent of studies through time, we correlated *Sample Extent* and the log(*Sample Extent* +1) with year. Both earthworm abundance and *Sample Extent* decreased over time (Figure A1). To account for this, we compared the results of models with and without using logged *Sample Extent* (+1) as a weighting in the models. Both models showed similar results (see results of the main text), suggesting that this change in sample extent through time has not affected our results.

a)

**
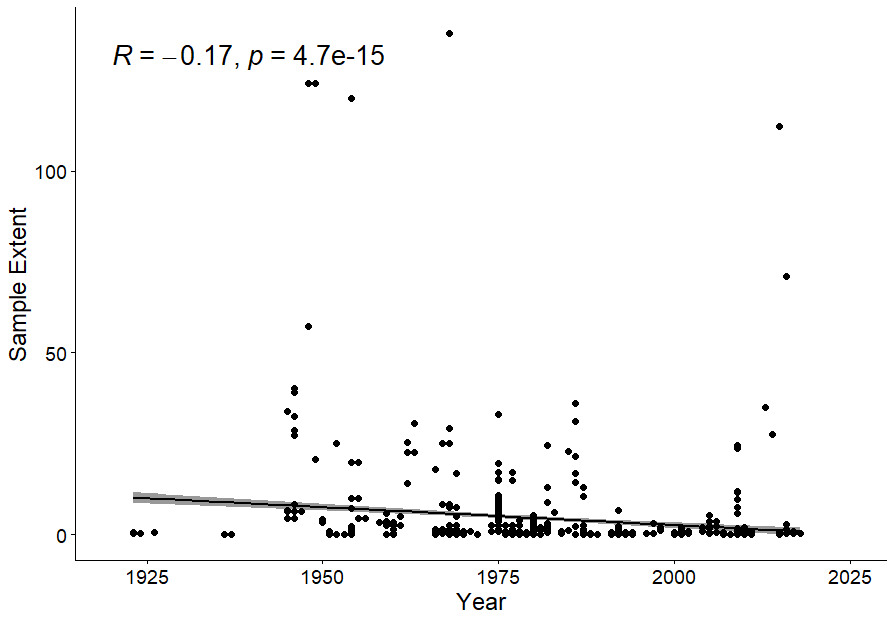
**

b)


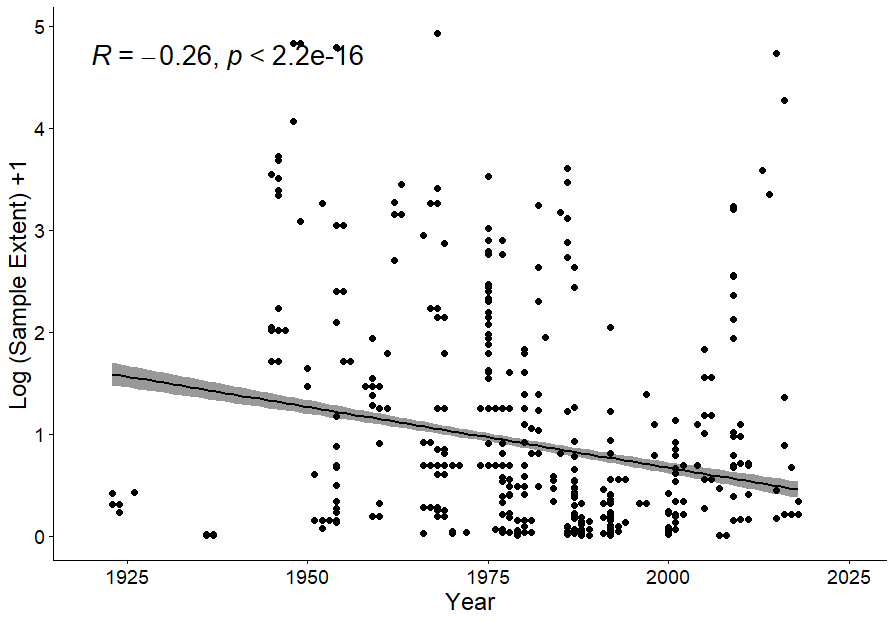


***Figure A1. The correlation*** *between year and a) Sample Extent and b) logged Sample Extent +1 (used in the weighted models).*

### Appendix 2: Testing for variation in the type of study published through time

It is possible that changes in the motivation of earthworm studies through time could alter the resulting data and bias our results. To test this, we split studies between those focussed on earthworms (1356 rows of data) compared to those focussed on other species, which also collected earthworm data (854 rows of data). Despite variation between the proportion of studies focused on earthworms through time (Figure A2), the inclusion of a variable *Focus* separating studies focussed on earthworms from “Other” studies, did not alter our results (compare Table A1 for Model 3 with Table 5).

***Table A1. Interaction Model 3 estimates*** *with the addition of the binary variable Focus (significant estimates are bold, stars represent: * <0.01, ** <0.001, *** <0.0001, SE = Standard Error).*

| **Variable Category** | **Variable** | **Unweighted** | | | **Weighted** | | |
| --- | --- | --- | --- | --- | --- | --- | --- |
|  |  | **Estimate** | **SE** | **P** | **Estimate** | **SE** | **P** |
|  | (Intercept) | **5.475** | **0.263** | **<0.001 (***)** | **5.658** | **0.262** | **<0.001 (***)** |
|  | year | **-0.018** | **0.006** | **0.005 (**)** | **-0.017** | **0.007** | **0.011 (*)** |
| *Broad_Habitat* | Human | 0.019 | 0.562 | 0.972 | -0.030 | 0.505 | 0.952 |
|  | Unenclosed | -0.502 | 0.277 | 0.069 (.) | **-0.950** | **0.183** | **<0.001 (***)** |
|  | Woodland | 0.621 | 0.360 | 0.085 (.) | 0.278 | 0.283 | 0.327 |
| *Season* | Multiple | **0.337** | **0.145** | **0.020 (*)** | **0.459** | **0.115** | **<0.001 (***)** |
|  | Spring | 0.039 | 0.061 | 0.521 | -0.008 | 0.059 | 0.896 |
|  | Summer | **-0.372** | **0.069** | **<0.001 (***)** | **-0.247** | **0.068** | **<0.001 (***)** |
|  | Winter | -0.030 | 0.073 | 0.677 | -0.053 | 0.078 | 0.502 |
| *Broad_Method* | Both | **-0.778** | **0.234** | **0.001 (***)** | **-0.744** | **0.209** | **<0.001 (***)** |
|  | Surface | **-1.316** | **0.159** | **<0.001 (***)** | **-1.302** | **0.157** | **<0.001 (***)** |
|  | Depth | **0.012** | **0.005** | **0.028 (*)** | 0.001 | 0.005 | 0.826 |
| *Focus* | Other | 0.468 | 0.279 | 0.094 (.) | 0.378 | 0.295 | 0.201 |
| *Broad_Habitat* Interactions | Human | -0.001 | 0.016 | 0.947 | -0.004 | 0.014 | 0.795 |
|  | Unenclosed | **0.021** | **0.010** | **0.045 (*)** | 0.009 | 0.008 | 0.263 |
|  | Woodland | **-0.027** | **0.010** | **0.009 (**)** | **-0.022** | **0.008** | **0.006 (**)** |

**
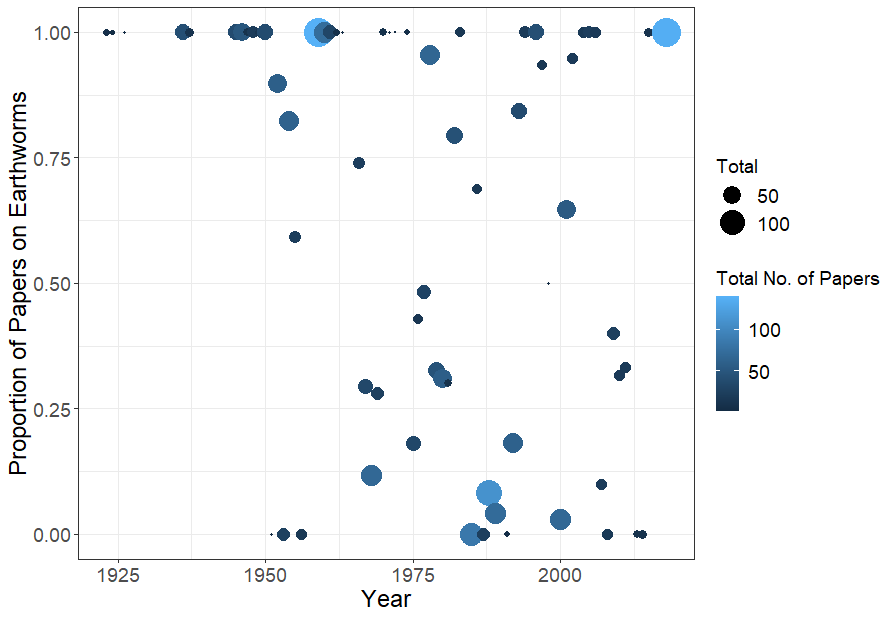
**

***Figure A2. Study focus.*** *The proportion of studies each year with a focus on earthworms, size and colour of point by total number of studies each year.*
