# Supplementary material for: Collation of a century of soil invertebrate abundance data suggests long-term declines in earthworms but not tipulids
Source: PLoS One. 2023 Apr 3;18(4):e0282069. doi: 10.1371/journal.pone.0282069 (PMC10069791; doi:10.1371/journal.pone.0282069)
Supplement: Attachment — Submitted filename: Response to Reviewers.docx [file pone.0282069.s004.docx]

**Response to Reviewers**

We would like thank the reviewers for their time and effort in reviewing our paper. As an early career researcher I found the comments really encouraging, and the reviewer’s suggestions were helpful and fair and we hope we have addressed each of them satisfactorily. All responses to the reviewers are in red and line numbers refers to the new submitted tracked changed copy of the manuscript, altered text appears in bold.

Thanks again for your time.

Reviewer #1: The case for doing the study of long-term abundance of earthworms and tipulids in the UK, as well as hypotheses, were very well stated. The study was well designed, and the authors took reasonable care to examine the appropriate papers and assemble a data set. The models developed and statistical methods used are well matched to the data and hypotheses to be tested. The manuscript is very well written and easy to understand.

Throughout the paper, every time I had a question, it was answered a sentence or two later, e.g. lines 411-413 about declining earthworm abundance in woodlands, and then on lines 417-420, the mechanism that I thought most likely responsible--declining soil moisture due to warmer summers, was discussed.

Potential problems due to the fact that the data do not result from standardized repeated sampling at the same locations over time, are very clearly explained, so that readers can make their own judgements about the validity of the study. The authors have taken all needed cautions and and used all appropriate adjustments to statistical methods to account for these potential shortcomings, and I believe the overall result is of significant value and a good advance in understanding of long-term changes in ecology, especially with respect to earthworms.

Suggestions for further research and ecological implications of declining earthworm populations, and management actions that may help to mitigate the problem are nicely stated at the end of the paper.

Thank you.

Reviewer #2: Review of “Collation of a century of soil invertebrate abundance data suggests long-term declines in earthworms but not tipulids”

Here the authors compile an impressive dataset of earthworm data in the UK and examine trends through time. As soil inverts are often overlooked, I liked the ambition of this paper and think it will have many readers and users of the dataset. I have lots of small comments rather than few big ones. The methods are not sufficiently explained at the moment, and the results are not always clearly presented. The stats need another look but I don’t expect the patterns to change. Overall it needs a bit of tidying, which I am confident the authors can do.

Comments (chronological rather than reflecting importance)
Line 51: “In other areas” – vague, I’d reword.

Line 51-53 changed to: “**For many other invertebrate groups**, a lack of robust monitoring data for many invertebrate groups has hampered our ability to detect long-term change [7]”.

Line 65-68 - Awkward sentence. Suggest rewrite.

Edited the sentence: “Whilst it is clearly not possible to go back in time and generate monitoring data ~~where there is none to answer this question~~, soil invertebrates have often been the subject of ecological study generating a potentially accessible record of historical soil invertebrate abundance data, that may be used to infer changes through time **[24]**.”

Line 67 – “Building on the approach of Robinson & Sutherland’ – I am not sure this statement is really fair to all the other authors of studies compiling datasets from data within past studies. It’s a very popular approach right now (e.g., van Klink et al., all the BioTime stuff, PREDICTS stuff etc.., without even getting into the core meta-analysis literature), so seems a bit unfair to only highlight one study of one the co-authors of the present study.

The Robinson & Sutherland study has particular similarities to ours by virtue of documenting long-term changes in weed seeds on farmland using a similar approach to the approach used here for soil invertebrates. However, recognising as the reviewer suggest that this is an approach now much more widely employed to generate large biodiversity datasets, we have edited Lines 69-73 to now read:

“Building on the approach of Robinson & Sutherland [25] who documented long-term declines in the availability of weed seeds on farmland from published studies**, and recognising recent developments in generating large biodiversity datasets from previously published studies (e.g. van Klink [3]),** we similarly attempt to identify potential long-term signals of changes in soil invertebrate abundance **from published studies**.”

Line 71 – not sure what “one” refers to – a country?

Yes, one is referring to the UK, changed to “in which” on Line 73.

Line 79- the inclusion of tipulids came as a bit of a surprise to me, as I wouldn’t regard them as obvious soil inverts. Maybe you want to say something about why you chose earthworms and tipulids for your study.

Actually many studies indicate that earthworms and tipulid larvae form the majority of soil macro-invertebrates in the soil, at least in the surface layers, and both groups combined are important components in the diet of avian predators (and of other taxa too). As a result, we have added this justification to Lines 79-82:

“**In doing so we focus on earthworms and tipulid larvae as the two main groups of soil macro-invertebrates in many UK soils (e.g. [32]) for which sufficient data were available for analysis, and that are also important components of the diet of many birds [16] and other taxa.”**

Line 80 – the hypothesis that trends vary between habitats is rather weak, without specifying how they vary. If you didn’t have any stronger prediction, then I would just drop this. Or just be frank and say you explored the differences between habitats.

Drawing on Burns et al. (2016) we have added “that those trends vary between habitats, **with the expectation for greater declines particularly in farmland, given the loss of farmland biodiversity in the UK [33]**” compared to other habitats to Lines 86-87.

Line 86 – Make clearer whether you searched for papers within a time frame

Added “, **see Table S1 for timeframe of searches**” to Line 96, referring to the PRISMA Checklist for dates of searches.

Line 106 - I would more clearly state your inclusion criteria – you say you “check if they contained relevant invertebrate data” but what would that look like. Data on abundance or biomass or xxx on earthworm (one or more species??) or tipulidae, in one or more years at one or more sampling sites, collected using standardized sampling methods across sites/years?? Its rather vague at the moment and so your search would not be repeatable.

Edited Lines 112-115: “Potentially relevant papers and theses were identified by title and then abstract (if present), and read to check if they contained relevant **and extractable/available species specific** invertebrate data **(i.e. they reported abundance or biomass data from locations and years)**.”, and added extra clarity in the Data first paragraph. On Lines 138-141 (and later) we explain that some data/studies were dropped if data was insufficient as this would not be possible to ascertain at the literature search stage.

Line 123 – I am not sure what you mean by “ correlations of these against ….”. Was this using the other studies that did present both total and adult numbers? Are you sure you used correlation? I can’t see how that would work – I could guess if you said regression. (Later, I see eqn 1 and 2, so it is regression). Please expand and clarify anyhow.

Changed Lines 131-136 to “**W**here **total abundance data were missing but** either adult numbers or wet biomass (for adults and/or immatures) data were provided alone, **total abundance was estimated from the provided data using regressions of total abundance against adult abundance (equation 1), and total abundance against biomass (equation 2) based on data from studies where both were presented (Fig S2 and S3).**”

Line 129 Density per m2 of soil sampled, I assume (but would that be a volume in m3?)? Or m2 land surface area of sampled? But then that wouldn’t account for sampling depth. Again, you just need to clarify a bit more.

Depth was accounted for in the models so it is abundance per m^2^ of soil, area rather than volume, as the abundance of earthworms vary with depth, i.e. are not randomly distributed with depth but in space. Added a sentence to Lines 143-144: “**As earthworm abundance varies with sample/soil depth, this was included in the models.**”

Line 134 – For this equation to be useful for others, we need to know the units of the wet biomass

Added “**(gm^-2^)”** to Line 148, also added “**(m^-2^)**” to Equation 1 and 2 on lines 145/146 and 148.

Line 131-134 – Based on this equation, I am guessing you did regression, and also guessing you assumed a normal distribution. For a response like ‘total number’, it would be more common to use a Poisson distribution to bound the predictions above zero. Did you consider this? Can you justify your current approach? Also was this a standard regression or did it include random effects? I can imagine that each study reported on multiple sample sites, so a study-level random effect (intercept and slope) would be warranted.

You are correct, these equations refer to the regressions used to calculate the missing values of earthworm/tipulid abundance from non-integer data. Once the mean of a Poisson distribution exceeds 30, then it approximates as a normal distribution. We were generating a calibration curve and are not interested in making inference at this stage, just in the predicted values to standardise all data. The R^2^ was also high (R^2^= 0.78 and 0.56, Lines 147 and 150) suggesting a good fit of the data.

The Analysis section of the paper details the models we used to analyse the data: negative binomial as the Poisson models were over dispersed see Lines 177-180. Random effects are detailed in Lines 180-183 (revised copy with track changes).

Line 157 - I think you mean that Year was centred so that 1960 was zero, so that the intercept of the fitted relationship represented the predicted abundance in 1960.

Yes, added to Line 174/175: “The intercept for *Year* was set at 1960, **so that the intercept of the fitted relationship represented the predicted abundance in 1960,**”

Line 158 – so were these data points just excluded from the analysis or included as an ‘other’ groups?

Excluded from the analysis as we stated in Line 176/177. It was mainly the fine method categories for the earthworm analysis and we couldn’t merge them as we excluded both chemical and core (e.g. core and orthodichlorobenzene) and surface (e.g. orthodichlorobenzene) methods, which were classed as different methods, ‘both’ or ‘surface’ in the broad methods categories so merging would be the same as the first analysis. This only excluded 3 studies and ~150 rows of data out of 2210. The tipulid habitats couldn’t be refined any further as we only had enough data for 2 broad habitat categories.

Line 160 – I am not quite sure what your response in your model was. Number or Number per m2?

“abundances (**per m^2^**)” edited on Line 155, edited in equation 1 and 2 on Lines 145 and 148 to “*Total* ***Abundance*** *of Earthworms* ***(m^-2^)***” for clarification.

I am thinking, based on line 139, it was the latter. But then I don’t understand how you modelled a decimal with negative binomial.

We rounded the values to whole numbers. Edited Line 155: “The abundances (per m^2^) of earthworms (A_E_) and tipulids (A_T_) **rounded to the nearest integer,**”. We also checked to see if a normal distribution was a better model, but the negative binomial was a much better fit using AIC.

I feel it would have been neater to have the number of individuals as the response, and then include sampling extent as an offset term. This would mean you keep a nice count variable as your response, but model the variable in sampling effort as an offset term (basically moving the denominator of the response to the right hand side of the equation). This is also much neater than doing any weighting since results from weighted model can be driven by a few studies given a large weight.
See this paper for an example
Dealing with Varying Detection Probability, Unequal Sample Sizes and Clumped Distributions in Count Data | PLOS ONE
<https://doi.org/10.1371/journal.pone.0040923>

We couldn’t use this method as some papers only published number of earthworms per m^2^/per yard^2^ etc. so we standardised all amounts of earthworms and tipulids to density per m^2^, so converting to counts and applying an offset would be effectively equivalent. Furthermore, some of the literature didn’t provide an area of sampling so where densities were reported the area was assumed to be either 1m, or 1 yard (= 0.9144m). In these cases, we assumed a weighting of 1 in order to be conservative, but we don’t feel that we could legitimately convert these values to absolute counts in the manner required to follow the Kotze method.

Edited sentence on Lines 141-143:

“**Given the variable nature of the data reported, with some studies presenting only densities and others total numbers, a**ll abundances were standardised to density per m^2^ for analysis.”

And we have added to Lines 192-199:

“**We followed the recommendations of Kotze et al. [40] by including information on seasonal variation in detectability, and by specifying the response variable as following a negative binomial distribution, as noted earlier. As we were modelling reported standardised densities rather than count data, due to the variable nature of the data (see earlier), we were unable to follow Kotze et al. [40] and use an offset with count (to give density), although the unweighted model results, based on density values, is equivalent to this approach. To capture any uncertainty we present both the weighted and unweighted** results**,** but for simplicity, most graphs present the non-weighted estimates.**”**

And edited Lines 566-570:

“Whilst we cannot treat our results as equivalent to trends from a structured, designed monitoring scheme**, as far as possible we have attempted to follow best practise with analyses of these data [40]**. **The** additional analyses **presented** do not indicate, at least for earthworms, that the putative **negative** earthworm trends reported, are subject to biases due to sample method, sample area/extent and study design, as best as we could measure them.”

Having said that, to assess the impact of this suggestion, we backcalculated the densities to abundances as best we could based on the information extracted from each paper using the area sampled as an offset – thus replicating as best we could the analysis suggested by the reviewer. This produced model outputs equivalent to those from the unweighted model, and so we are happy that the approach that we have taken in the analysis is in line with the reviewer’s suggestion as far as we can. There remains value in also presenting the results of the weighted model for the reasons explained in the paper, as this gives greater weight to the results from studies that are likely to be most representative because they have the greatest coverage / sample size.

Table 2 – where did the habitat info come from? Did you overlay the study coordinates onto a land cover map? Or did you try to infer based on the study description of its habitat.

Based on description from the study – see Lines 118-122 where we detail the information extracted from the literature and added “**description of** habitat type” to Lines 119/120 to make this clearer.

Line 188 – What was the median time span and number of sampling years across studies? Also the mean and IQR of sampling sites per study?

For the earthworm data the median time span was 1 years, and the maximum time span is 13 years from one study. 52% of studies only collected data in a single year, 31% of studies collected data over 2 years 14% of studies collected data over 3 and 4 years and there were only 2 longer term studies (11 and 13 years), which varied in location, method, habitat etc. The median time span per study for the Tipulids was 2 therefore 2 year studies, and the maximum 26 years, but again this was only one study, and 75% of the studies contained data in one or 2 years, the majority were single year studies.

Added a sentence to Lines 212-214: **“The median time span per earthworm study was 1 year and 2 years for tipulids (and the longest study 13 and 26 years, respectively), most were single year studies.”**

Line 213 – For the coefficients of factors of multiple levels, I am not sure what is being compared with what. E.g., line 213 Fewer earthworms were collected in summer compared with when? I am guessing Autumn since that is the season missing in Table 3. If so, any reason why you compared each season to Autumn in particular (apart from it being the R default!)? Similar comment applies for the other factors here like habitat and method. You don’t mention any post-hoc multiple comparisons – which is fine. But you still need to justify your reference levels for comparison for each variable e.g., the reference level could be the most common level or it could be one where you hypothesize high or low values.

Summer and winter are sensitive to weather so the base could be either Autumn or Spring, Autumn was chosen as numbers are more stable and comparable across years. Habitat base was set to farmland, as we anticipated it would have the lowest values and we stated that farmland was the base in Line 249, and method was set to core as this was the most common and reliable method of sampling. Edited Lines 239-241:

“Focussing on the unweighted model, there were significantly fewer earthworms **in samples** in the summer **than the autumn**, and using chemical (Surface) and both surface and core samples combined **than by using cores**,”.

Added a note to the caption of Table 2 Line 200: “***Base factor is noted first for each variable.****”*, we have also added this information to all table captions.

Figure 2 is missing some regression lines

Figure 2 is only displaying the spread of the data over time before analysis – the predict function was used to show the decline from the model in Figure 3, this was not shown for tipulids as there was no significant trend found. We have added the calibration graphs to the Supplementary Material.

Table 5 – bit unclear what numbers are being presented in this table. Are these the interaction term coefficient? Or the trends within each subset? but this wouldn’t naturally fall out the model unless you removed the intercept. Again, if the former, make the reference levels clear.

Yes, this Table represents the model output from the model with the interaction term of habitat and year to get the habitat specific trends, which are at the bottom of the table. This is straight out of the model summary and the *year* estimate is the trend in farmland. We removed the intercept to plot the trends but present the raw estimates in the tables which compare to the base, farmland.

Added a sentence to Table 5 caption Line 315: “***compared to the base, farmland, trend shown in the year estimate****”*.

Figure axes labels of “Estimate” aren’t very informative. Be more specific.

These figures show the estimate of trend from the model, we have changed the y axis labels of Figure 6-8 to “Annual Change”.

It would be nice to see Fig 3 with study-level random slopes for year to get a better idea of whether the overall mean is really a consistent pattern at the study level too. This plot could go in the SI. The overall mean decline isn’t massively convincing looking at the data points here! If anything, it looks non-linear – but that might be caused by between-study differences, which is why I suggest the random slopes.

Unfortunately, there isn’t much point plotting study-level slopes as the majority are single year studies which will be plotted as one point, from which we can’t calculate a trend or get/plot a slope.

Added to Lines 551-553: **“(only one study had repeat samples through time that spanned more than a decade, whilst at the 10-km square level, data were recorded from multiple years spanning over 10-years from only 10 squares)”**

I think Outhwaite’s paper (ref 14) also included tipulidae – how do your results compare with hers? Worth a mention.

Thank you for pointing this out, we have now added this reference to Lines 381-384: “**Based on analyses of occurrence data, there is no evidence for strong trends in an index of occupancy of craneflies in the UK from 1970-2015 [14], in line with our conclusions for changes in tipulid abundance.”**

And to Lines 403-404: **“, and mixed trends in the occurrence of many terrestrial insect groups [14]).**” And to Line 521: **“, matching occurrence trends since the 1970s [14].”**

Literature
Key papers are not cited, including some very high-profile ones on earthworm data and distribution. It would only be fair to these authors who already compiled a large database of earthworm data to cite them:
Phillips, H.R.P., Bach, E.M., Bartz, M.L.C. et al. Global data on earthworm abundance, biomass, diversity and corresponding environmental properties. Sci Data 8, 136 (2021). <https://doi.org/10.1038/s41597-021-00912-z>
Phillips et al. Global distribution of earthworm diversity, Science, 371, 6525, (2021). /doi/10.1126/science.abe4744

Thank you for bringing these papers to our attention, we have now cited the former reference in Line 68. We note that our dataset is from a longer time period (1926 cf. 1973).

**Journal Requirements:**

When submitting your revision, we need you to address these additional requirements.

1. Please ensure that your manuscript meets PLOS ONE's style requirements, including those for file naming. The PLOS ONE style templates can be found at 
<https://journals.plos.org/plosone/s/file?id=wjVg/PLOSOne_formatting_sample_main_body.pdf> and 
<https://journals.plos.org/plosone/s/file?id=ba62/PLOSOne_formatting_sample_title_authors_affiliations.pdf>

Done.

2. We note that you have stated that you will provide repository information for your data at acceptance. Should your manuscript be accepted for publication, we will hold it until you provide the relevant accession numbers or DOIs necessary to access your data. If you wish to make changes to your Data Availability statement, please describe these changes in your cover letter and we will update your Data Availability statement to reflect the information you provide.

3. Please amend either the abstract on the online submission form (via Edit Submission) or the abstract in the manuscript so that they are identical.

4. We note that [Figures # 1a and 1b] in your submission contain [map/satellite] images which may be copyrighted. All PLOS content is published under the Creative Commons Attribution License (CC BY 4.0), which means that the manuscript, images, and Supporting Information files will be freely available online, and any third party is permitted to access, download, copy, distribute, and use these materials in any way, even commercially, with proper attribution. For these reasons, we cannot publish previously copyrighted maps or satellite images created using proprietary data, such as Google software (Google Maps, Street View, and Earth). For more information, see our copyright guidelines: <http://journals.plos.org/plosone/s/licenses-and-copyright>.
 
We require you to either (1) present written permission from the copyright holder to publish these figures specifically under the CC BY 4.0 license, or (2) remove the figures from your submission:
 
   1. You may seek permission from the original copyright holder of Figures # 1a and 1b to publish the content specifically under the CC BY 4.0 license.  
 
We recommend that you contact the original copyright holder with the Content Permission Form (<http://journals.plos.org/plosone/s/file?id=7c09/content-permission-form.pdf>) and the following text:
“I request permission for the open-access journal PLOS ONE to publish XXX under the Creative Commons Attribution License (CCAL) CC BY 4.0 (<http://creativecommons.org/licenses/by/4.0/>). Please be aware that this license allows unrestricted use and distribution, even commercially, by third parties. Please reply and provide explicit written permission to publish XXX under a CC BY license and complete the attached form.”
 
Please upload the completed Content Permission Form or other proof of granted permissions as an "Other" file with your submission.
 
In the figure caption of the copyrighted figure, please include the following text: “Reprinted from [ref] under a CC BY license, with permission from [name of publisher], original copyright [original copyright year].”
 
   2. If you are unable to obtain permission from the original copyright holder to publish these figures under the CC BY 4.0 license or if the copyright holder’s requirements are incompatible with the CC BY 4.0 license, please either i) remove the figure or ii) supply a replacement figure that complies with the CC BY 4.0 license. Please check copyright information on all replacement figures and update the figure caption with source information. If applicable, please specify in the figure caption text when a figure is similar but not identical to the original image and is therefore for illustrative purposes only.
The following resources for replacing copyrighted map figures may be helpful:
 
USGS National Map Viewer (public domain): <http://viewer.nationalmap.gov/viewer/>
The Gateway to Astronaut Photography of Earth (public domain): <http://eol.jsc.nasa.gov/sseop/clickmap/>
Maps at the CIA (public domain): <https://www.cia.gov/library/publications/the-world-factbook/index.html> and <https://www.cia.gov/library/publications/cia-maps-publications/index.html>
NASA Earth Observatory (public domain): <http://earthobservatory.nasa.gov/>
Landsat: <http://landsat.visibleearth.nasa.gov/>
USGS EROS (Earth Resources Observatory and Science (EROS) Center) (public domain): [http://eros.usgs.gov/#](http://eros.usgs.gov/)
Natural Earth (public domain): <http://www.naturalearthdata.com/>

**All map data are open source and inserted** *“Contains Ordnance Survey data © Crown Copyright and database right 2022.”* **Into Figure 1 caption.**

5. Please include captions for your Supporting Information files at the end of your manuscript, and update any in-text citations to match accordingly. Please see our Supporting Information guidelines for more information: <http://journals.plos.org/plosone/s/supporting-information>.

We have now included the Supporting Table/Figure labels and Titles at the end of the manuscript.
